# A new record of odd-scaled snake (Serpentes, Xenodermidae) from Vietnam: expanded description of *Parafimbrios vietnamensis* based on integrative taxonomy

**DOI:** 10.3897/zookeys.1048.66477

**Published:** 2021-07-08

**Authors:** Nikolai L. Orlov, Oleg A. Ermakov, Tao Thien Nguyen, Natalia B. Ananjeva

**Affiliations:** 1 Zoological Institute, Russian Academy of Sciences, Universitetskaya nab. 1, St. Petersburg, 199034, Russia Zoological Institute, Russian Academy of Sciences St. Petersburg Russia; 2 Penza State University, Krasnaya ul. 40, Penza, 440026, Russia Penza State University Penza Russia; 3 Vietnam National Museum of Nature, Vietnam Academy of Science and Technology, 18 Hoang Quoc Viet Road, Cau Giay, Hanoi, Vietnam Vietnam National Museum of Nature, Vietnam Academy of Science and Technology Hanoi Vietnam

**Keywords:** Distribution, molecular identification, morphology, odd-scaled snake, *
Parafimbrios
*, Xenodermidae, Vietnam

## Abstract

Based on the combination of molecular and morphological data, we herein report the second known finding of the xenodermid snake species *Parafimbrios vietnamensis* Ziegler, Ngo, Pham, Nguyen, Le & Nguyen, 2018. The male individual was found in the Yen Bai Province of northwestern Vietnam, more than 200 km from the type locality in Lai Chau Province. Genetic divergence between the newly-collected male and the holotype was low (1.7%), and is in agreement with morphological data that supports that they are conspecific. We give a detailed description of the morphological characters and coloration of the new record and provide an expanded diagnosis of *P. vietnamensis*. *Parafimbrios* is a poorly-understood genus, and our recent discovery brings the total number of known specimens of the genus to nine, 1/3 of them having been found in Vietnam (one specimen of *P. lao* and now two specimens of *P. vietnamensis*).

## Introduction

**Figure 1.**
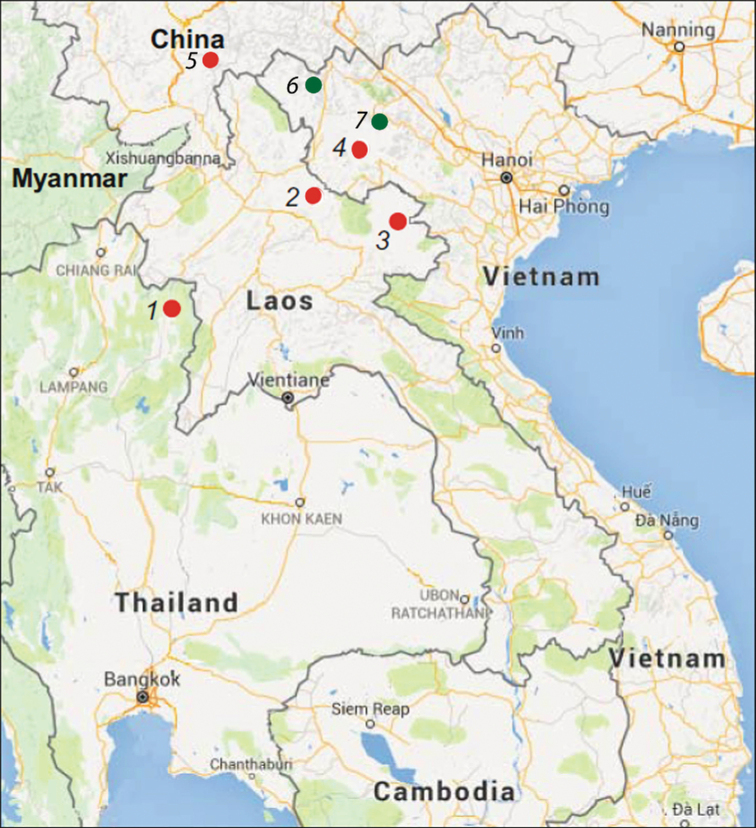
Map of Indochina showing the known localities of *Parafimbrios lao* (red dots) and *P. vietnamensis* (green dots) (after Teynié et al. 2015; Nguyen et al. 2015; Teynié and Hauser. 2017; Ziegler et al. 2018; Bo et al. 2020) ***1*** Pua district, Nan Province, Thailand ***2*** Ngoi District, Luangphrabang Province, Laos (type locality of *P. lao*) ***3*** Vieng Xai District, Houaphan Province, Laos ***4*** Thuan Chau District, Son La Province, Vietnam ***5*** Jiangcheng County, Yunnan Province, Southwestern China; *P. vietnamensis****6*** Hoang Ho Village, Phang So Lin Commune, Sin Ho District, Lai Chau Province, Vietnam, 22.4167°N, 103.3681°E (type locality of *P. vietnamensis*) ***7***ZISP 31426, Che Tao Village, The Mu Cang Chai Species and Habitat Conservation Area (SHCA), Yen Bai Province, Vietnam, 21.5435°N, 104.0364°E (new record of *P. vietnamensis*).

The snake family Xenodermidae is one of the most poorly-known groups of Asian reptiles. The family is composed of five genera and 23 recognized species that are distributed throughout South, Southeast, and East Asia (Uetz et al. 2020). Many species are only known from a single or a few specimens from a limited number of localities. Among these five genera, *Parafimbrios* Teynié, David, Lottier, Le & Vidal, 2015 only has two recognized species, which are endemic to Indochina and occur across Laos, Vietnam, Thailand and south China (Nguyen et al. 2015; Teynié et al. 2015; Teynié and Hauser 2017; Ziegler et al. 2018; Cai et al. 2020) (Fig. 1).

The genus was originally described as monotypic, including only *Parafimbrios lao* Teynié, David, Lottier, Le & Vidal, 2015 from Louangphabang and Houaphan provinces in northeastern Laos, based on a unique set of morphological characters and high levels of genetic divergence (Teynié et al. 2015). Three years later, the second species was described as *Parafimbrios vietnamensis* Ziegler, Ngo, Pham, Nguyen, Le & Nguyen, 2018 from Lai Chau Province in northwestern Vietnam. Until recently, *P. vietnamensis* was only known from the holotype male. However, during a recent herpetological survey in Yen Bai Province in northern Vietnam, another male specimen was collected that had the unique set of morphological characteristics and color pattern typical for *P. vietnamensis*. Subsequent molecular analysis confirmed the morphological findings and we herein provide a detailed description of the second known specimen of *P. vietnamensis* and an expanded diagnosis of the species.

This discovery brings the total number of known specimens of *Parafimbrios* to nine and of *P. vietnamensis* to two. This work highlights the difficult nature of discovering specimens of this snake family and the need for further surveys in these areas of Vietnam and Eastern Laos.

## Material and methods

This study is based on a single male specimen of *Parafimbrios vietnamensis* (ZISP 31426) from Che Tao Village, Che Tao Commune, of the Mu Cang Chai Species and Habitat Conservation Area (SHCA) (21.5435°N, 104.0364°E, elevation 1300 m) in Yen Bai Province, Vietnam. The specimen was collected on November 30^th^, 2019, by Nikolai Orlov and Larissa Ioganssen and was fixed and subsequently stored in 75% ethanol. A tissue sample was preserved separately in 95% ethanol. The specimen was deposited in the herpetological collection of the Zoological Institute, Russian Academy of Sciences (ZISP), St. Petersburg, Russia.

### Morphological examination

Sex was determined by inspection of the presence or absence of hemipenes. Measurements were taken to the nearest mm with digital calipers. Paired meristic characters are given as left/right. The methodology of measurements and meristic counts followed Teynié et al. (2015) and Ziegler et al. (2018). The following characters and ratios were evaluated and calculated (Table 1): snout-vent length measured from tip of snout to anterior margin of cloaca (SVL); tail length measured from posterior margin of cloaca to tip of tail (TaL); total length, corresponding to SVL + TaL (TL); ratio of tail length to total length (TaL/TL); number of maxillary teeth, counted by investigating the right maxilla for teeth/sockets. The pholidosis characters taken or counted are as follows: dorsal scale rows counted at one head length behind head, at midbody, and at one head length before vent, respectively. The number of ventrals, subcaudals, supralabials, infralabials, suboculars, loreals, preoculars, postoculars, temporals and cloacal scales were counted. Morphological comparisons were based on data from Teynié et al. (2015), Nguyen et al. (2015), Teynié and Hauser (2017), Ziegler et al. (2018), and Cai et al. (2020).

**Table 1.** Measuremements (in mm), dentition and scalation of *Parafimbrios vietnamensis* compared to *P. lao*.

Characters	*Parafimbrios lao* male holotype MNHN 2013.1002	*P. lao* male not collected	*P. lao* male TBU PAR.127	*P. lao* male QSMI 1381	*P. lao* male QSMI 1382	*P. lao* male not collected	*P. lao* female CIB2019090746	*Parafimbrios vietnamensis* male holotype IEBR A.2018.7	*P. vietnamensis*ZISP 31426 subadult male
Country	Laos	Laos	Vietnam	Thailand	Thailand	Thailand	China	Vietnam	Vietnam
Province	Luangphrabang	Houaphan	Son La	Nan	Nan	Nan	Yunnan	Lai Chau	Yen Bai
Snout-vent length mm	236	298	310	294	333	?	256	222	298
Tail length (TaL) mm	49	55	56.5	66	~72	?	53	44	56
Total length (TL) mm	285	353	366.5	360	~405	~350	309	266	354
TaL / TL	0.172	0.156	0.150	0.183	0.177		0.171	0.165	0.163
Maxillary teeth	27	-	-	-	-	-	-	27	27
Dorsal scale rows	27–25–23*	27–25–23*	27–27–25	25	25?	25?	29-27-24	**35–33–29**	**31-35-27**
Ventrals	177+2	189+1	185	179	171+	?	168	**164**	172+2
Subcaudals	56	55	53	61	?	?	52	**49**	**48**
Cloacal	1	1	1	-	-	-	-	1	1
Supralabials	8/8	7/7	8/8	-	-	-	-	8/8	8/8
Infralabials	8/8	7/7	8/7	-	-	-	-	7/7	7/7
Subocular	1/1	1/1	1/1	1/?	1/?	1/?	1/1	1/1	1/1
Loreal	1/1	1/1	1/1					1/1	1/1
Preocular	1/1	1/1	1/1	1/1	1/?	1/?	1/1	1/1	1/1
Postoculars	2/2	2/2	2/2	2/2	2/?	2/?	2/2	2/2	2/2
Temporals	2+2/2+2	2+2/2+1	2+2/2+2-	-	-	-	-	**4+4/4+5**	2+3/2+3
Source	Teynié et al. 2015	Teynié et al. 2015	Nguyen et al. 2015	Teynie, Hauser 2017	Teynie, Hauser 2017	Teynie, Hauser 2017	Cai et al. 2020	Ziegler et al. 2018	Our data

Museum abbreviations are as follows:

**ZISP**Zoological Institute, Russian Academy of Sciences, St. Petersburg, Russia;

**IEBR** Institute of Ecology and Biological Resources, Vietnamese Academy of Science and Technology, Hanoi, Vietnam;

**MNHN**Muséum National d’Histoire Naturelle, Paris, France;

**TBU PAR** Faculty of Biology and Chemistry, Tay Bac University, Son La Province, Vietnam;

**QSMI** Queen Saovabha Memorial Institute, Thai Red Cross Society, Bangkok, Thailand;

**CIB**Museum of Herpetology, Chengdu Institute of Biology, Chinese Academy of Sciences, Chengdu, China.

### Molecular data and phylogenetic analyses

Molecular data were generated for the specimen reported herein from Yen Bai Province, Vietnam. Homologous sequences were obtained from GenBank. DNA was extracted using the standard salt-extraction method (Aljanabi and Martinez 1997), combined with lysis by proteinase K. The cytochrome *c* oxidase subunit 1 (*COI*) gene fragment (660 bp) was amplified using the primer pair UTF 5'-TGT AAA ACG ACG GCC AGT TCT CAA CCA AYC AYA ARG AYA TYG G-3' and UTR 5'-CAG GAA ACA GCT ATG ACT ARA CTT CTG GRT GKC CRA ARA AYC A-3', following the protocol of Lissovsky et al. (2010). PCR products were cleaned by elution with a concentrated saline solution from 6% polyacrylamide gel. Sequencing was performed using an ABI 3500 automatic sequencer (Applied Biosystems) and BigDyeTerminator 3.1 kits 103 (Applied Biosystems). The nucleotide sequence was aligned with the BioEdit software (Hall 1999) and further edited manually. The final sequences were deposited in GenBank (MW542529).

We combined the sequence of the new specimen of *Parafimbrios* reported here with nine sequences downloaded from GenBank. We selected two outgroups, *Xenopeltis unicolor* Reinwardt, 1827 AB179620, *Acrochordus granulatus* Schneider, 1799 AB177879 (Dong and Kumazawa 2005), and five species for our ingroup, *P. vietnamensis* Ziegler, Ngo, Pham, Nguyen, Le & Nguyen, 2018 MH884515 (Ziegler et al. 2018), *P. lao* Teynié, David, Lottier, Le & Vidal, 2015 KT374005 (Nguyen et al. 2015), KP410746, *Fimbrios klossi* Smith, 1921 KP410744–45, *Xenodermus javanicus* Reinhardt, 1836 KP410747 (Teynié et al. 2015), and *Achalinus spinalis* Peters, 1869 MK064591 (Peng et al. 2017; Li et al. 2020) of the family Xenodermidae (Table 2). We used MEGA v. 7.0. (Kumar et al. 2016) for phylogenetic analyses using the Maximum Likelihood (ML) method. The HKY+G+I model was selected as the most appropriate DNA substitution model for the dataset using jModelTest 2.1.10 (Posada 2008). Minimum evolution (ME) and neighbor-joining (NJ) analyses were used as a complement to our maximum likelihood analyses. Node support was estimated using 1000 bootstrap replicates. Lastly, we calculated uncorrected pairwise divergences between all samples in MEGA v. 7.0.

**Table 2.** In-group samples used in molecular analyses.

Species name	GenBank No.	Locality	Voucher	Reference
*Parafimbrios lao*	KP410746	Louangphabang Province, Laos	MNHN 2013.1002	Teynié et al. 2015
*Parafimbrios lao*	KT374005	Son La, Vietnam	TBU PAR.127	Nguyen et al. 2015
*Parafimbrios vietnamensis*	MH884515	Lai Chau, Vietnam	IEBR A.2018.7	Ziegler et al. 2018
*Parafimbrios vietnamensis*	MW542529	Yen Bai, Vietnam	ZISP	This study
*Fimbrios klossi*	KP410745	Gia Lai, Vietnam	IEBR A.2013.56	Teynié et al. 2015
*Fimbrios klossi*	KP410744	Quang Ngai Province, Vietnam	IEBR 3275	Teynié et al. 2015
*Xenodermus javanicus*	KP410747	Maninjau Lake, Sumatera Barat Province, Sumatra, Indonesia	–	Teynié et al. 2015
*Achalinus spinalis*	NC032084	Shaanxi, China	HS12093	Peng et al. 2017
*Xenopeltis unicolor*	AB179620	–	NUM-Az0378	Dong, Kumazawa 2005
*Acrochordus granulatus*	AB177879	–	NUM-Az0375	Dong, Kumazawa 2005

## Results and discussion

### Description of the second specimen from Vietnam (Figs 2, 3).

**Figure 2.**
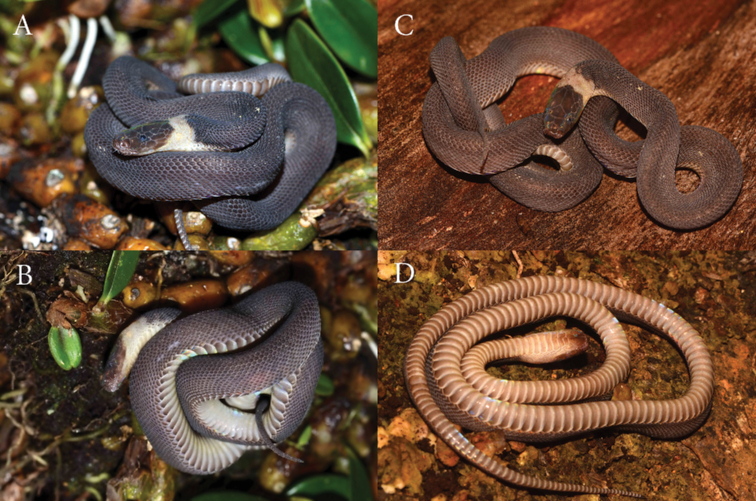
*Parafimbrios vietnamensis*ZISP 31426 **A** dorsal view in life **B** ventrolateral view in life **C** dorsal view in life **D** ventral view of the preserved specimen.

**Figure 3.**
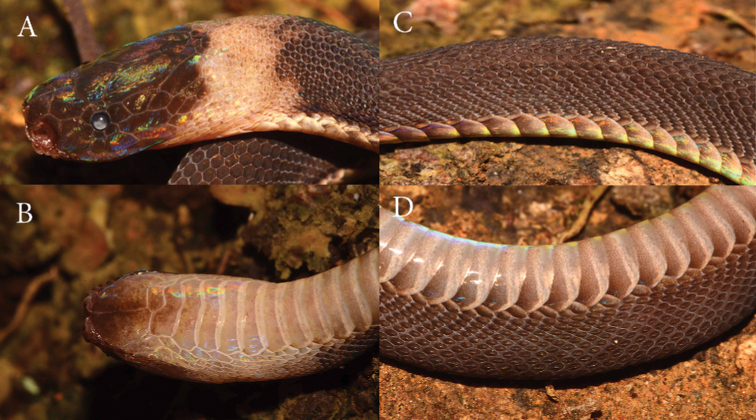
*Parafimbrios vietnamensis*ZISP 31426 in life **A** dorsal view of head **B** ventral view of head **C** dorsolateral view of midbody **D** ventrolateral view of midbody.

Morphological characters of the second male specimen are concordant with those in the original description of *Parafimbrios vietnamensis* by Ziegler et al. (2018). The specimen has a cylindrical and slender body (Figs 2, 3). Head not distinct from neck, dorsally covered with large shields; eyes middle-sized, with a vertically sub-elliptic pupil.

Snout-vent length 298 mm; tail length 56 mm; total length 354 mm; ratio of tail length to total length 0.163. Dorsal scale rows 31-35-27; laterally rounded ventral scales 172+2; subcaudals 48; postoculars 2/2; preoculars 1/1; suboculars 1/1; supralabials 8/8; infralabials 7/7 (Fig. 3A, B). Rostral triangular, its upper edge separating it from the internasals; nasal in contact zone with rostral with curved raised edge; suture between the internasals much longer than that between the prefrontals; 8 supralabials, the first four bearing raised edges; temporals 2+3/2+3 (Fig. 3A, B); 48 unpaired subcaudals; total length 354 mm; tail length 56 mm; TaL/TL ratio 0.163.

Dorsal scales small, cycloid, keeled from region behind the neck onwards, every second scale of outermost row distinctly enlarged; 31 scales around the anterior part of the body; two dorsal scale rows corresponding to a ventral plate; distinct, laterally-rounded ventrals; single subcaudal scales; cloacal shield entire (Figs 2D, 3C, D). Left hemipenis basally everted.

Morphological data of this new specimen, the holotype of *P. vietnamensis*, and comparative data on seven specimens of *P. lao* are summarized in Table 1. The second male specimen from Vietnam differs from the male holotype of *P. vietnamensis* in having a larger size (SVL 298 mm vs. 222 mm), a higher number of ventrals (172+2 vs. 164), and fewer temporals (2+3/2+3 vs. 4+4/4+5). The pholidosis characteristics of the number of ventrals and temporals look more similar to those seen in *P. lao*.

**Phylogenetic analysis.** Molecular analyses corroborate the morphological data. The new specimen is significantly genetically divergent from *P. lao* by at least 10%. The new sample was strongly supported as the sister lineage to the holotype of *Parafimbrios vietnamensis* (bootstrap support = 100%) (Fig. 5). Genetic divergence between the newly-collected specimen and the holotype was low (1.7%), and thus supports that they are conspecific. The mean uncorrected pairwise genetic distance between the two species within the genus *Parafimbrios* was 7.7±0.8%.

**Coloration.** The color of the dorsum is gray-brown, with varying intensity of brown depending on the angle of the light, and with iridizing sequins. The head is separated from the neck by a wide, light-colored nuchal collar extending to the ventral surface and to the chin; the collar does not completely cover the ventral part of the chin but stops at the ventral scales. The dorsal surface of the head is reddish-brown from the lateral edge of the head to the parietal scale and frontal scales. The gular region is brown; the two huge mandibular plates are brown anteriorly, lightening posteriorly. The color of the nuchal collar is a light cream, slightly white-pinkish. The color of the belly is smoky gray with lightened lateral edges of the abdominal scales.

**Distribution.***Parafimbrios vietnamensis* is only known from two provinces (Lai Chau Province, Yen Bai Province) in northwestern Vietnam (Fig. 1). The second species, *P. lao*, is only known from northern Vietnam in the Son La Province, but has a much wider distribution in Laos, Thailand, Vietnam and southern China. This area of Vietnam is the highland region associated with Hoang Lien Son ridge (Lai Chau, Lao Cai, and part of Yen Bai). Biogeographically, the species inhabits the south-eastern part of the Sikang-Yunnan floristic Province of the Holarctic floristic kingdom (Takhtajan 1978).

**Figure 4.**
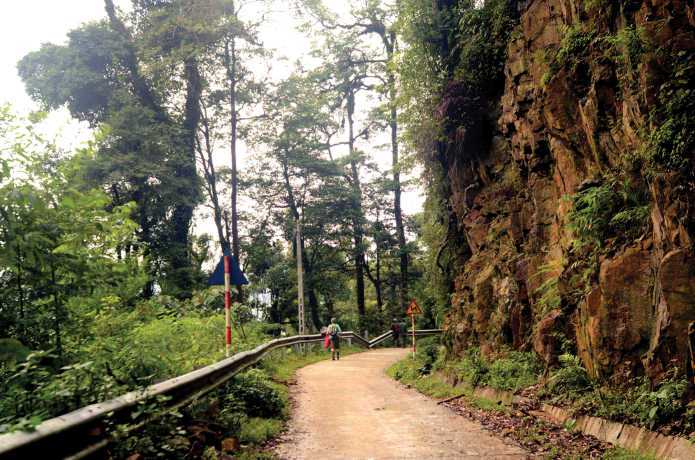
Habitat of *P. vietnamensis* in the primary polydominant forest in Yen Bai Province, Vietnam.

**Ecology and habitat.** The specimen was collected on November 30, 2019 after an overnight rain, at midnight. The specimen was found in the leaf litter in primary polydominant forest (Fig. 4) at an elevation of 1300 m a.s.l.

The second male specimen from Vietnam differs from the male holotype of *P. vietnamensis* in having a larger size (SVL 298 mm vs. 222 mm), a higher number of ventrals (172+2 vs. 164), and fewer temporals (2+3/2+3 vs. 4+4/4+5). The number of ventrals and temporals are similar to those reported from *P. lao*. Morphological data from our new finding, the holotype of *P. vietnamensis*, and comparative data for seven specimens of *P. lao* are summarized in Table 1.

Due to the only minor morphological differences between the holotype of *Parafimbrios vietnamensis* and the new specimen described in this paper, and to the very low genetic divergence, we consider the new specimen conspecific with the holotype of *P. vietnamensis*. The expanded diagnosis of the species is as follows:

A species of the genus *Parafimbrios*, characterized by the following combination of characters: 1) rostral laterally with two raised, curved edges; the upper one, together with a horizontal curved ridge of tissue, separates the rostral from the internasals; 2) nasal in contact zone with rostral with curved, raised edge; 3) nasal in contact zone with supralabials with two small oblique, curved raised edges located above first and second as well as above second and third supralabials; 4) suture between the internasals much longer than that between the prefrontals; 5) supralabials 8, the first four bearing raised edges; 6) infralabials 7; mental and anterior three to four infralabials with raised edges; 7) temporals 2+3.2+3–4+4–5; 8) (31–35)-(33–35)-(27–29) dorsal scale rows; 9) laterally rounded ventrals 164–172+2; 10) unpaired subcaudals 48–49; 11) total length at least 266–354 mm in males (with a tail length of 44–56 mm and a TaL/TL ratio of 0.16). The coloration corresponds to that given in the original description (Ziegler et al. 2018): brownish-black above, with a broad yellow neckband widening towards the venter and stretching to the chin region; dorsal head surface in part reddish-brown, in particular in the middle of the parietals and frontal; venter grayish-brown, lighter anteriorly, darker towards tail region; ventrals anteriorly and laterally darker. It should be noted that we are most likely dealing with subadult specimens, with a pronounced whitish-pinkish nuchal collar. For example, in the related species *Parafimbrios lao*, the nuchal collar dims and disappears with age, which can be seen in Teynié et al. (2015: fig. 4). More findings of this rare snake will add information to this species’ diagnosis.

*Parafimbrios vietnamensis* was previously known only from Hoang Ho Village, Phang So Lin Commune, Sin Ho District, Lai Chau Province in northern Vietnam (Ziegler et al. 2018). Although the new locality in Yen Bai Province is more than 200 km southeast of the type locality, the samples exhibit a relatively low amount of genetic divergence from each other.

**Figure 5.**
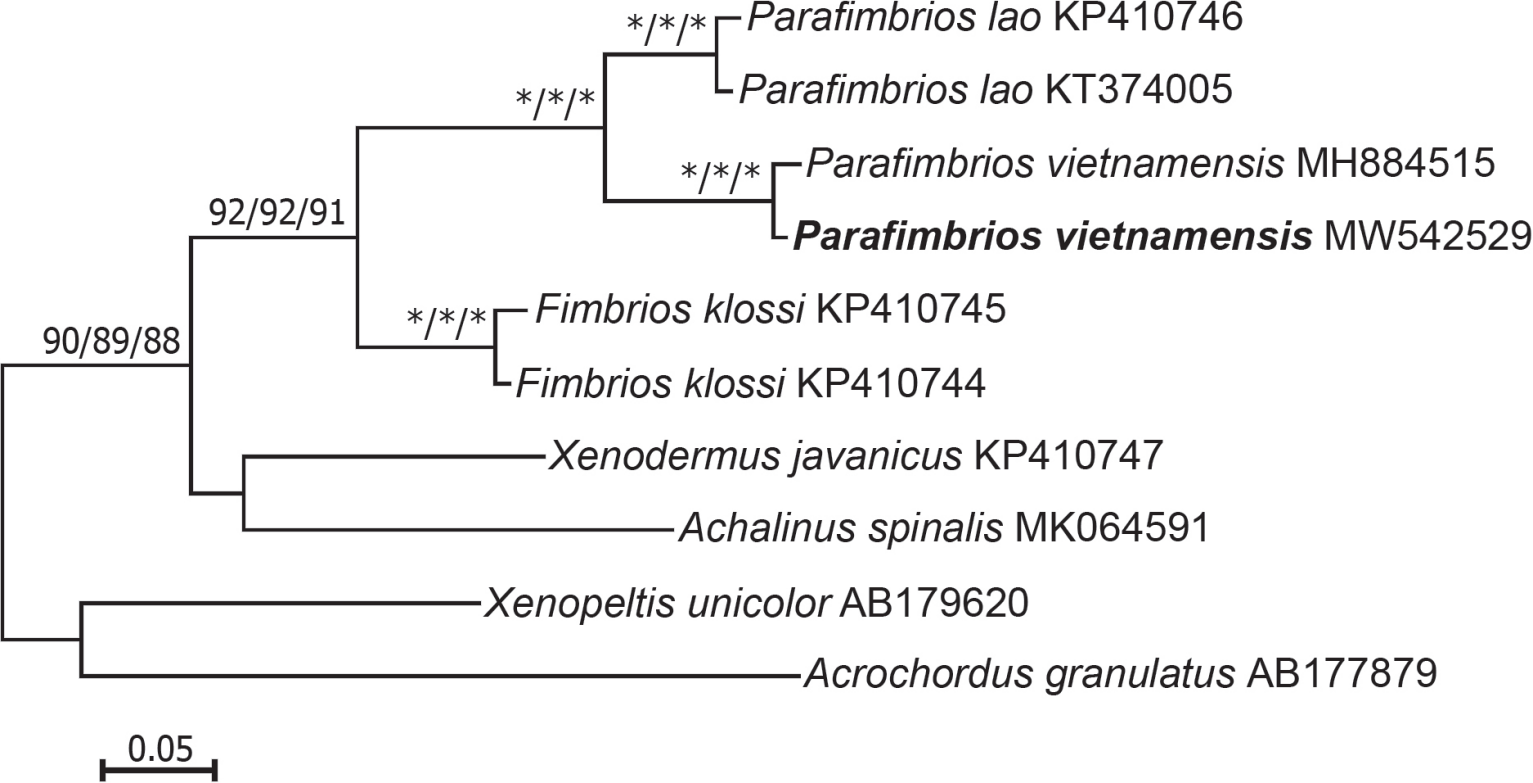
ML phylogenetic tree (HKY+G+I model) based on the mitochondrial *COI* gene. Numbers near the branches denote percentage bootstrap resampling support from 1000 replications (ML/ME/NJ). Bootstrap support is only shown for values exceeding 80%. Asterisks represent 100% values. Scale bar shows substitutions/site.

The mountain systems in this area are composed of magmatic silicate rocks, particularly granite and quartzite, which were formed as extensive intrusions of late Paleozoic and Mesozoic age (Dovzikov et al. 1965). Tertiary tectonic movements uplifted these mountain terrains up to the present-day elevations, and subsequent erosion processes formed the present-day characteristic rocky landscape of this highland area, with very steep slopes, numerous rocky cliffs and deep and narrow river canyons (Fridland 1961; Averyanov et al. 2003). This area is extremely humid, with warm rainy summers and cold foggy and misty winters and without a distinct dry period. The peak of rainfall arrives in the summer months. Morning dew is very common throughout the mountain zone, as well as frequent heavy fog. Humid, cold, northeast monsoon winds that bring heavy fog, mist and drizzle are very common in the winter and early spring (Averyanov et al. 2003). Zonal types of vegetation in the studied area belong to a group of closed evergreen tropical monsoon (seasonal) submontane forests (Averyanov et al. 2003).

There are now three known specimens of genus *Parafimbrios* recorded in Vietnam: one of *P. lao* and two of *P. vietnamensis*. The discovery highlights the difficulty of finding specimens of Xenodermidae and the need for further surveys in these areas of Vietnam and Eastern Laos.

So far, the following snake species were reported from Che Tao Village, Che Tao Commune, Mu Cang Chai District, Yen Bai Province in Vietnam: *Oreocryptophis porphyraceus* (Cantor, 1839), *Hebius bitaeniatus* (Wall, 1925), *H. boulengeri* (Gressitt, 1937), *Pararhabdophis chapaensis* Bourret, 1934, *Sinonatrix percarinata* (Boulenger, 1899), *Pareas hamptoni* (Boulenger, 1905) (Le et al. 2018). This new record of the rare snake species *Parafimbrios vietnamensis* is an important supplement to the list of snakes recorded from Yen Bai Province, and highlights its conservation needs.
